# Infectious Toscana Virus in Seminal Fluid of Young Man Returning from Elba Island, Italy

**DOI:** 10.3201/eid2804.211920

**Published:** 2022-04

**Authors:** Giulia Matusali, Alessandra D’Abramo, Chiara Terrosi, Fabrizio Carletti, Francesca Colavita, Francesco Vairo, Gianni Gori Savellini, Claudia Gandolfo, Gabriele Anichini, Eleonora Lalle, Licia Bordi, Angela Corpolongo, Micaela Maritti, Luisa Marchioni, Maria Rosaria Capobianchi, Concetta Castilletti, Maria Grazia Cusi, Emanuele Nicastri

**Affiliations:** National Institute for Infectious Diseases “Lazzaro Spallanzani” IRCCS, Rome, Italy (G. Matusali, A. D’Abramo, F. Carletti, F. Colavita, F. Vairo, E. Lalle, L. Bordi, A. Corpolongo, M. Maritti, L. Marchioni, M.R. Capobianchi, C. Castilletti, E. Nicastri);; Siena University Hospital, Siena, Italy (C. Terrosi, G.G. Savellini, C. Gandolfo, G. Anichini, M.G. Cusi)

**Keywords:** Toscana virus, meningitis/encephalitis, viral isolation, seminal fluid, viral persistence, genital tropism, viruses, vector-borne infections, Italy

## Abstract

We report detecting infectious Toscana virus in the seminal fluid of a 25-year-old man from Italy returning from Elba Island. The presence of infectious virus in human semen adds Toscana virus to the long list of viruses detected in this genital fluid and indicates a potential for sexual transmission.

Toscana virus (TOSV) is an arthropodborne virus, belonging to the genus *Phlebovirus*, that was first isolated in Monte Argentario in 1971 from the sand flies *Phlebotomus perniciosus* and *P. perfiliewi* (*1*,*2*). Some years later, TOSV was detected in the cerebrospinal fluid of 2 patients with meningitis (*3*,*4*), confirming its role in the etiology of this neurologic disorder. To date, phylogenetic analysis has distinguished 3 genotypes of TOSV (lineages A, B, and C), which are differentially distributed in the countries of the Mediterranean Basin (*5*). TOSV A is the most common cause of summer viral meningitis in central Italy and France and has a frequency eclipsing that of enteroviruses (*6*); lineage B is present in France and Spain, and lineage C is present in Croatia and Greece (*7*). Seroprevalence studies in TOSV-endemic regions indicate that most infections result in mild or self-limited febrile illnesses, whereas neurologic disorders develop in a small percentage of infected persons (*6*). We report the case of a young man with TOSV meningitis and prolonged persistence of TOSV in blood and semen.

## The Study

On October 5, 2019, a 25-year-old man in otherwise healthy condition was admitted to the emergency department of Aurelia Hospital (Rome, Italy), for a history of acute headache, mental confusion, dysarthria, and high-grade fever since October 1. He had been living in Italy since January 27, 2019, after a 4-year period of residence in London, UK, where he abused alcohol and cocaine. During April 23–October 1, 2019, he worked as a waiter in a hotel on Elba Island, Tuscany, where he lived with his girlfriend. Their most recent sexual intercourse occurred on September 25. He reported several mosquito bites related to use of a shower located outside the hotel.

Physical examination revealed signs of meningeal irritation in the absence of focal neurologic deficits. Results of chest radiograph were negative; blood tests showed unremarkable liver and renal function results but indicated lymphocitopenia (total lymphocytes 760/μL), and thrombocytopenia (platelet count 72,000/μL).

Brain computed tomography excluded brain swelling with evidence of elevated intracranial pressure. Examination of cerebrospinal fluid (CSF) revealed pleocytosis (88 cells/μL) consisting primarily of polymorphonuclear cells, normal glucose levels, and increased total protein levels (129 mg/dL). PCR results were negative for neurotropic pathogens (*Neisseria meningitides*, *Streptococcus pneumoniae*, *Escherichia coli* K1, *Haemophilus influenza*, *Listeria monocytogenes*, *S. agalactiae*, herpes simplex virus types 1 and 2, varicella zoster virus, cytomegalovirus, enterovirus, human parechovirus, human herpesvirus 6, and *Cryptococcus neoformans/gattii*).

The patient was transferred to the intensive care unit of the National Institute for Infectious Diseases “Lazzaro Spallanzani” (INMI) in Rome in a state of psychomotor agitation. Orotracheal intubation was performed with mechanical ventilation because of worsening Glasgow Coma scale score (10). Empiric treatment with acyclovir, ceftriaxone, ampicillin, and steroids was started. Epidemiologic evaluation of the case (i.e., season and residence in a TOSV-endemic area) suggested the possibility of an arboviral disease.

**Figure 1:**
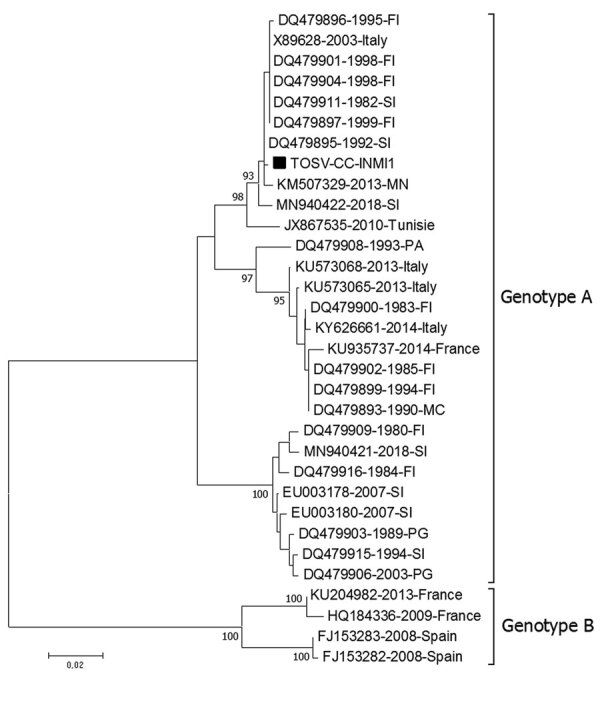
Phylogenetic tree based on the partial medium segment of Toscana virus (TOSV) (black square) (nucleotide position 2178–2742 of TOSV reference sequence X89628.1) detected in cerebrospinal fluid of young man returning from Elba Island, Italy. Tree was built using the neighbor-joining method and evolutionary distances computed by using the Kimura 2-parameter method. The rate variation among sites was modeled with a gamma distribution (shape parameter = 0.3). Each record consists of accession number, year, and place of detection/isolation. TOSV genotypes A and B are indicated. Phylogenetic analysis was conducted in the MEGA7 software package (http://www.megasoftware.net). FI, Florence; MC, Macerata; MN, Mantua; PA, Palermo; PG, Perugia; SI, Siena.

Real-time reverse transcription PCR (rRT-PCR) for TOSV (in-house rRT-PCR method targeting the viral medium RNA segment) was positive on CSF collected 5 days after symptom onset (cycle threshold 30.82); molecular assays for Naples, Sicilian, and Cyprus virus and flaviviruses and culture of CSF for bacteria and fungi were negative. Sequencing analysis of the medium gene revealed that the TOSV RNA detected in CSF belonged to lineage A (Figure 1; name: TOSV-CC-INMI1). Specific IgM (1:320) and IgG (1:640) titers were evaluated by indirect immunofluorescence assay (sandfly fever virus Mosaic 1; EUROIMMUN, https://www.euroimmun.com) on serum collected 17 days after symptom onset.

After clinical improvement, the patient was extubated on October 10; 2 days later, he was transferred to the high-isolation unit. Results of magnetic resonance imaging of the brain were unremarkable. The patient was discharged on October 19 in good clinical condition, and a 6-month follow-up examination did not reveal any sequelae.

**Figure 2:**
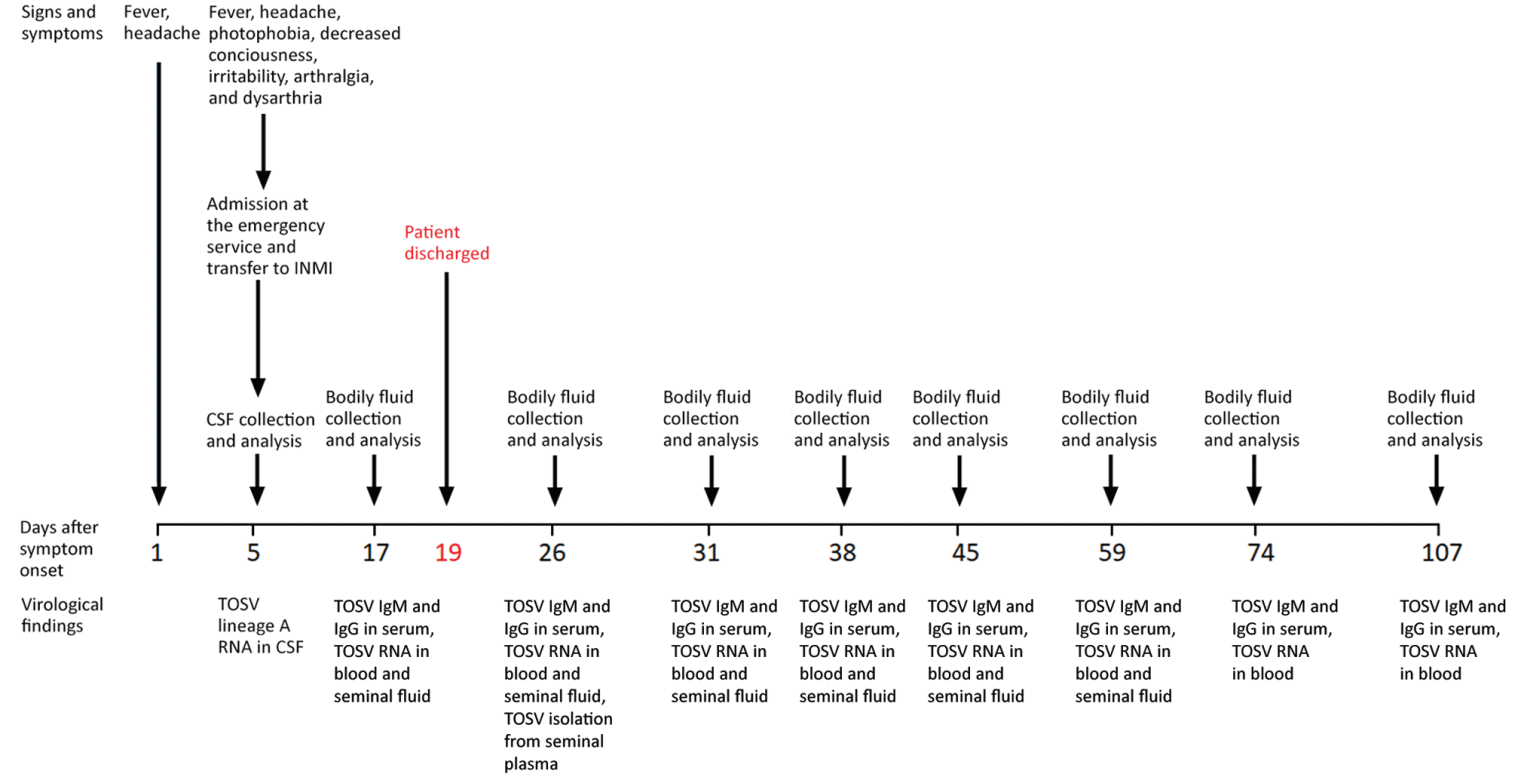
Clinical and laboratory findings during acute phase and follow-up treatment of man with infectious TOSV detected in seminal fluid, Italy. CSF, cerebrospinal fluid; INMI, National Institute for Infectious Diseases “Lazzaro Spallanzani”; TOSV, Toscana virus.

After signing a written informed consent and upon INMI Ethical Board approval (issue: 14/2015), the patient was enrolled in a study on the tropism of arboviruses. To this purpose, every 7 or 14 days, and according to the patient’s willingness and ability to reach the INMI, we collected whole blood, serum, saliva, urine, and seminal fluid for viral RNA and specific antibody detection (Figure 2).

Serologic tests demonstrated the presence of both TOSV IgM and IgG from day 17 to the end of the follow-up period at day 107 after symptom onset (Table 1). Afterward, no more visits were scheduled because of the start of the coronavirus disease pandemic. Although TOSV RNA was never detected in serum, saliva, or urine, rRT-PCR results showed an unusual long-term persistence of low-level viremia for >3 months (Table 2).

**Table 1:** TOSV IgM and IgG titer in serum samples of man returning from Elba Island, Italy*

Day after symptom onset	IgM	IgG
17	320	640
26	320	1,280
31	320	640
38	320	1,280
45	640	1,280
59	160	320
74	320	640
107	320	640

*Titer expressed as reciprocal of serum dilution.

**Table 2:** C_t_ values of Toscana virus RNA detected in bodily fluids of man returning from Elba Island, Italy*

Day after symptom onset	Serum	Whole blood	Urine	Saliva	Seminal fluid	Spermatozoa	Round cells
17	ND	32.21	Neg	Neg	24.26	27.58	35.14
26	ND	32.06	Neg	Neg	24.20	NT	NT
31	ND	33.94	Neg	Neg	26.13	NT	NT
38	ND	35.11	Neg	Neg	28.80	NT	NT
45	ND	35.31	Neg	Neg	31.65	NT	NT
59	ND	35.04	Neg	Neg	38.20	NT	NT
74	ND	36.00	Neg	Neg	Neg	NT	NT
107	ND	38.08	Neg	Neg	Neg	NT	NT

*C_t_, cycle threshold; ND, not detected by reverse transcription PCR; neg, negative; NT, not tested.

Of note, viral RNA was detected in seminal fluid from day 17 until day 59 after symptom onset. Results of rRT-PCR on seminal cells demonstrated that TOSV RNA was associated with both spermatozoa and round cells fractions, separated by gradient at the time of collection (PureSperm 40/80; NidaCon International AB, https://www.nidacon.com) (Table 2).

To determine the potential for viral transmission from semen, we inoculated Vero cells (ATCC CCL-81) grown in cell vials with blood, seminal fluid, and seminal plasma collected at day 26 after symptom onset. We were able to isolate TOSV from seminal plasma when the culture showed a cytopathic effect at passage 3. We confirmed TOSV positivity by immunofluorescence on infected cells using anti-nucleocapsid protein serum (data not shown). We extracted total RNA from supernatant of infected cells and amplified the viral genome (*8*), then sequenced the full-length small and medium segment and part of the large segment by the Sanger method for molecular confirmation (accession nos. MZ643219 for small complete coding sequence [CDS], MZ643217 for medium complete CDS:, and MZ643218 for large partial CDS).

## Conclusions

Most human TOSV infections are asymptomatic or appear as a nonspecific febrile disease. Neuroinvasive disease can occur, and, although self-resolving in most cases, TOSV infection of the central nervous system can be severe (*9*). In this study, we describe unusual long-term TOSV viremia and detection and persistence of TOSV in human semen. We could not recover infectious TOSV from blood samples, probably because of high levels of specific antibodies; the persistent low-level viremia could also derive from residual virus in the cellular components of blood. 

We detected viral RNA in both acellular and cellular fractions of semen and isolated infectious TOSV from seminal plasma. Moreover, we detected the viral RNA in both spermatozoa and round seminal cells; both might include potential targets of TOSV infection. Nevertheless, the exact tropism for the genital tract and the origin of TOSV in semen remain to be elucidated. The patient reported no symptoms of genital inflammation, despite persistence of TOSV in genital secretions and the observation of sperm abnormalities (scarce mobility of spermatozoa was observed throughout the study period). To date, there have been few reports on genital involvement in men infected by TOSV; reported manifestations have included testicular pain, orchitis, and epididymitis (*10*–*14*). The finding of TOSV in human semen puts TOSV in the long list of viruses detected in this fluid (*15*), illuminating the potential for sexual transmission as an alternative route of viral spread. In this case, we did not find evidence of TOSV sexual transmission to the patient’s partner, whose serum, blood, saliva, urine, and vaginal fluid never tested positive for TOSV RNA. Moreover, despite the presence of specific IgM, we observed no IgG seroconversion during the 3-month follow-up. All these observations suggest tropism of TOSV for the male genital tract that warrants further investigation.
